# BACTIBASE second release: a database and tool platform for bacteriocin characterization

**DOI:** 10.1186/1471-2180-10-22

**Published:** 2010-01-27

**Authors:** Riadh Hammami, Abdelmajid Zouhir, Christophe Le Lay, Jeannette Ben Hamida, Ismail Fliss

**Affiliations:** 1 STELA Dairy Research Center, Nutraceuticals and Functional Foods Institute, Université Laval, G1K 7P4 Québec, QC, Canada; 2 Unité de Protéomie Fonctionnelle & Biopréservation Alimentaire, Institut Supérieur des Sciences Biologiques Appliquées de Tunis, Université El Manar, Tunisie

## Abstract

**Background:**

BACTIBASE is an integrated open-access database designed for the characterization of bacterial antimicrobial peptides, commonly known as bacteriocins.

**Description:**

For its second release, BACTIBASE has been expanded and equipped with additional functions aimed at both casual and power users. The number of entries has been increased by 44% and includes data collected from published literature as well as high-throughput datasets. The database provides a manually curated annotation of bacteriocin sequences. Improvements brought to BACTIBASE include incorporation of various tools for bacteriocin analysis, such as homology search, multiple sequence alignments, Hidden Markov Models, molecular modelling and retrieval through our taxonomy Browser.

**Conclusion:**

The provided features should make BACTIBASE a useful tool in food preservation or food safety applications and could have implications for the development of new drugs for medical use. BACTIBASE is available at http://bactibase.pfba-lab-tun.org.

## Background

The dramatic rise in antibiotic-resistant pathogens has renewed efforts to identify, develop and redesign antibiotics. Bacteriocins are non-toxic inhibitors of bacteria and thus represent potential alternatives or complements to conventional antibiotics in the treatment of infections and in livestock production.

Bacteriocins were first identified almost 100 years ago. A heat-labile substance in *Escherichia coli *V culture supernatant was found toxic to *E. coli *S and given the name "colicin". It was thus decided that bacteriocins would be named after the producing species [1]. Fredericq demonstrated that colicins were proteins and that the inhibitory activity depended on the presence of specific receptors on the surface of sensitive cells and was therefore limited to specific species or strains [2]. Since then, bacteriocins have been found among most families of bacteria and many actinomycetes and described as universally produced, including by some members of the Archaea [3,4]. Klaenhammer estimates that 99% of all bacteria probably produce at least one bacteriocin and the only reason we have not isolated more is that few researchers are looking for them [5].

Two main features distinguish the majority of bacteriocins from conventional antibiotics: bacteriocins are ribosomally synthesized and have a relatively narrow killing spectrum (3). They make up a highly diverse family of proteins in terms of size, microbial target, mode of action and release and mechanism of immunity and can be divided into two broad groups: those produced by Gram-negative bacteria and those produced by Gram-positive bacteria [6,7].

We have previously developed and described a database (BACTIBASE) that contains calculated or predicted physicochemical properties of bacteriocins produced by both Gram-positive and Gram-negative bacteria [8]. BACTIBASE is a relational database that uses the MySQL server with a web interface composed of several PHP, JavaScript, Perl and Python scripts. The relational design of the database (i.e. the tables and the relations between them) has since been updated. In this paper, we describe this and other modifications, in particular the expansion of the biological information and the improvement of the query and navigation interfaces. We have also integrated several applications and utilities for bacteriocin sequences analysis and characterization. The new features should make BACTIBASE an even more useful tool in food preservation or food safety applications and could have implications for the development of new drugs for medical use.

## Construction and content

The content and format of BACTIBASE have been described previously [8]. While the general format has remained essentially unchanged, data retrieval and presentation have been improved. Data collection and annotation was done essentially the same way as for version 1 and the dataset is currently limited to natural sources. All microbiological information was collected from the literature by PubMed search. A physicochemical dataset was designed using SciDBMaker [9] and then provided with empirical formula, mass, length, isoelectric point, net charge, the number of basic, acidic, hydrophobic and polar residues, hydropathy index, binding potential index, instability index, aliphatic index, half-life in mammalian cells, yeast and *E. coli*, cysteine and glycine content, extinction coefficient, absorbance at 280 nm, absent and most prevalent amino acids, secondary (α-helix or β-strand) and tertiary structure (when available), physical method used for structural determination (e.g. NMR spectroscopy or X-ray diffraction) and critical residues for activity, whenever information was available. The Jmol applet http://www.jmol.org was included for tertiary structure visualization. The statistical interface provides data on peptide sequence, function and structure. Data were analyzed using SPSS software (version 17, SPSS Inc.) and medians and standard deviations were calculated. The following is a brief description of the database content.

**Figure 1:**
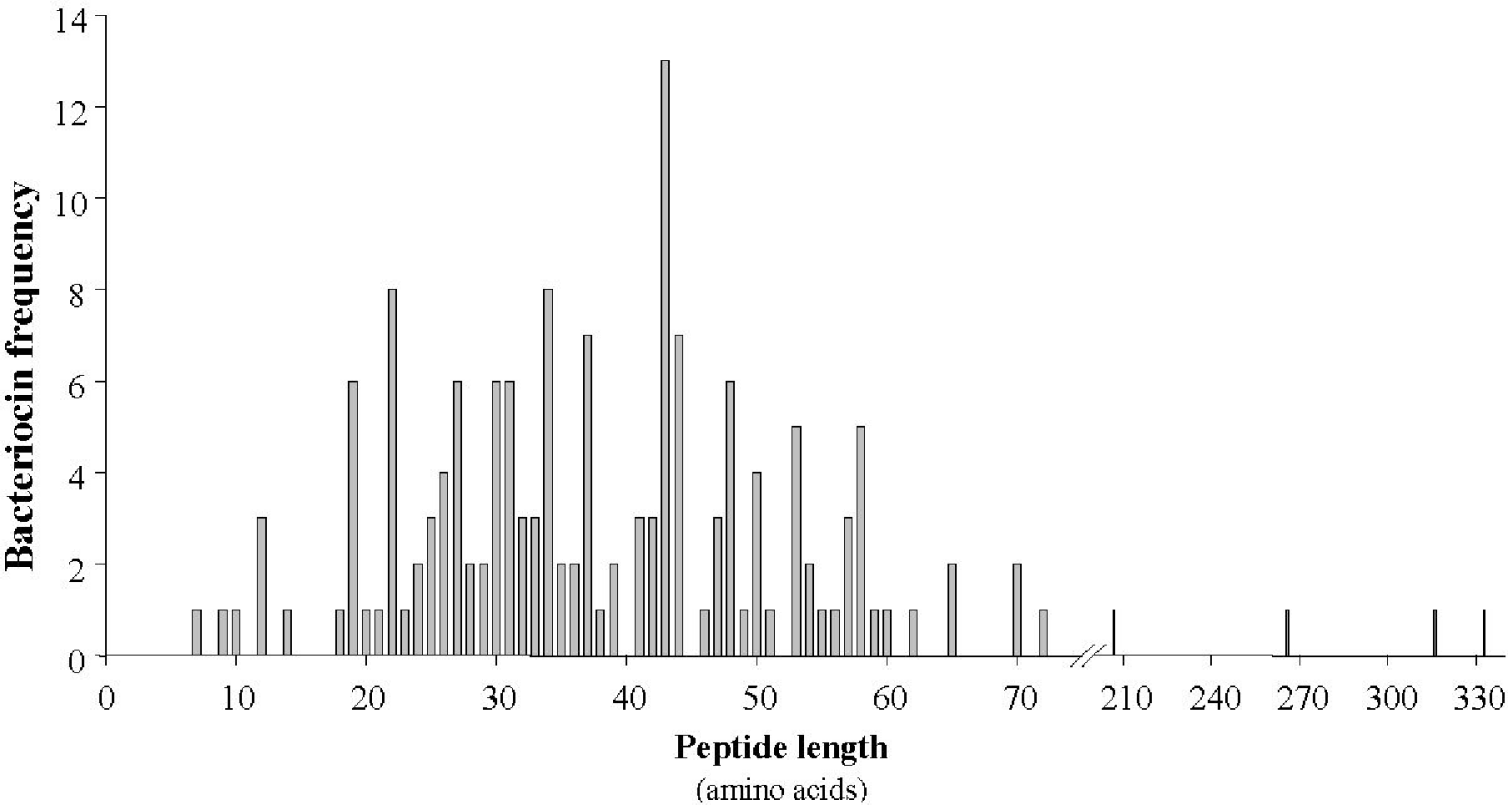
**Peptide length distribution among the bacteriocins produced by the Gram-positive organisms in the BACTIBASE database**.

The current release of the BACTIBASE dataset (version 2, July 2009) contains 177 (44% more) bacteriocin sequences, of which 156 are the products of Gram-positive organisms and 18 of Gram-negative organisms. We also note the presence of three bacteriocins from the Archaea domain. The database now comprises 31 genera, as shown in Table S1 (additional file 1). Without surprise, the lactic acid bacteria (order *Lactobacillales*) make up the predominant group of producers, with 113 bacteriocins. Figure 1 illustrates the distribution of peptide length among the bacteriocins of Gram-positive organisms, which varies from 20 to 60 amino acids in 84% of cases. In contrast, Gram-negative bacteriocins come in a very broad range of lengths, the longest (BAC127) being 688 amino acid residues (data not shown). Amino acid percentages are close to those calculated for the previous version of BACTIBASE. Table S1 lists averages for the net charge and amino acid contents of the bacteriocins produced by each of the 31 genera. These characteristics may serve as a physicochemical fingerprint for each group. Investigation of the PDB database revealed only 22 bacteriocins having a resolved 3D structure (by NMR spectroscopy or crystallography). Some of these are represented by more than one structure in the PDB database, bringing the total number of known 3D structures to 40. BACTIBASE provides detailed statistics on the bacteriocins. The improved database should be useful for discovering and characterizing potent bacteriocins or designing novel peptides with greater antimicrobial activity against pathogens.

## Utility

### Taxonomy explorer

**Figure 2:**
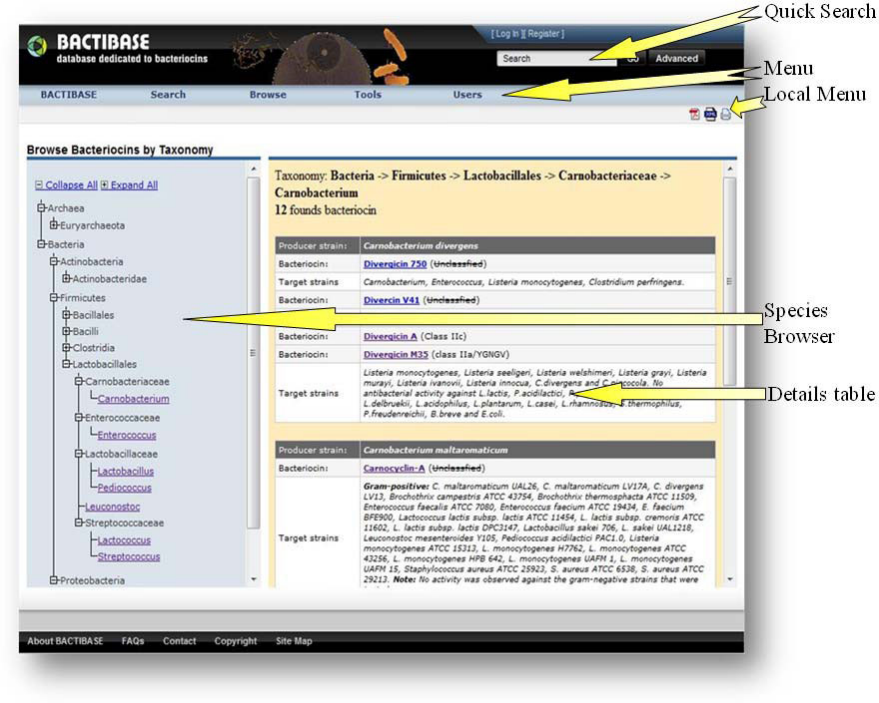
**The user interface displaying the taxonomic browser**.

An integrated phylogenetic tree view was designed (Figure 2) to facilitate data retrieval via bacterial species name. The tree is displayed on the left and the corresponding bacteriocins are listed in tabled form on the right. In the default setting, the tree is collapsed and displays only the phyla assigned to the Bacteria and Archaea domains along with a brief definition of these in the table. A click on the 'Expand All' hyperlink at the top displays the entire tree and the user may then select and expand the branches by clicking on nodes (family/genus). All bacteriocins associated with the selected genus are summarized in the table and a report can be generated in PDF format for further analysis. Clicking on the provided link displays the detailed entry for each bacteriocin.

### References sub-database

The entire database is linked to the Bibliography section, which lists all published scientific articles consulted on the subject of each bacteriocin. The 'news' link points to the latest hundred published review articles in PubMed.

### Bacteriocin structural analysis tool set

Several useful tools for protein analysis have been integrated into the platform. Users may search bacteriocin homologies using not only the BLAST program [10] but also FASTA [11] and SSEARCH [11]. Multiple sequence alignment may be done using CLUSTALW [12], MUSCLE [13] and T-COFFEE [14] and displayed graphically using the embedded JalView applet [15]. We used hidden Markov modeling (HMM) to produce bacteriocin profiles for each known family. The HMMER program was used to provide statistical descriptions of family consensus sequences [16] in order to allow users to identify the bacterial family that produces the bacteriocins most similar to their sequences.

Understanding of the molecular function of bacteriocins has been enhanced greatly by insight gained from three-dimensional structure. During the past decade, the use of homology modeling to study protein structure has become widespread. This technique generates a model of a protein using an experimental structure of a related protein as a template. We thus incorporated the program MODELLER [17] into the platform, which implements comparative protein structure modeling by satisfaction of spatial restraint. A sub-database of bacteriocins for which experimental structures have been developed was built. Users should note that only bacteriocins are used as templates in the homology modeling process. A modeling pipeline has been developed for automatic homology modeling from an initial bacteriocin sequence. This feature should be very useful for the *in silico *design of novel bacteriocins. The ability to develop novel bacteriocin-based-drugs that target prokaryotic as well as eukaryotic cells may open new possibilities for the design of improved antibiotics possessing refined characteristics.

### Linking to the BACTIBASE database

It remains very easy to link directly to a specific BACTIBASE entry. With our new domain name, users may link directly to records using their BACTIBASE ID in the format http://bactibase.pfba-lab-tun.org/bacteriocinsview.php?id=BAC059, which will allow links to be maintained even if the bacteriocin data changes.

### Forum

The forum section is provided to allow anyone to exchange information or ask questions regarding bacteriocins.

## Discussion

### Comparison with other databases

In the last decade, several databases dealing with AMPs were developed http://bactibase.pfba-lab-tun.org/links.php. The AMSDb (see: http://www.bbcm.univ.trieste.it/~tossi/amsdb.html), ANTIMIC [18], APD2 [19], and CAMP [20] databases cover all AMPs sequences from diverse origins. Alternatively, some databases focus on AMPs produced by bacteria (BACTIBASE [8]), plants (PhytAMP [21]) and shrimp (PenBase [22]). While AMSdb database covers only AMPs of eukaryotic origin, ANTIMIC database contains about 1700 AMPs from diverse origins (eukaryotes, prokaryotes). Regrettably, this resource was discontinued. The Antimicrobial Peptide Database (APD2) is the most popular of the currently available public collections (containing 944 antibacterial peptides of eukaryotic and prokaryotic origin) [19]. Recently, a new database containing a large Collection of Anti-Microbial Peptides (CAMP) was developed and holds 3782 antimicrobial sequences [20]. While lantibiotics are the class I of bacteriocins, the CAMP database lists them as a distinct family from bacteriocins. This may confuse novice users. Although APD2 and CAMP databases contain very general information about peptides of all types having antibacterial, antifungal or antiviral activities and originating from either eukaryotic or prokaryotic cells, bacteriocins are not described with a useful amount of detail in either of these databases. Not only does BACTIBASE (version 2, July 2009) contain significantly more antimicrobial peptides of bacterial origin, than the APD2 and CAMP databases (177 in BACTIBASE versus ~120 in APD2 and ~68 in CAMP), but also every entry in BACTIBASE is much more detailed. BACTIBASE features, for example, physicochemical and structural information, detailed lists of target organisms and a description of the mode of action for each bacteriocin -- data not available in APD2 or any other online resource (to the best of our knowledge). Also, BACTIBASE hosts a rich and highly usable collection of references, where (i) each entry has been supplied with a short annotation summarizing its topic in ~10 words or less, (ii) is cross-linked to PubMed, and (iii) can be conveniently exported to Citation Manager Software of user's choice. The database provides several tools for bacteriocin sequence analysis (unavailable in APD2; unavailable or static in CAMP), such as homology search, multiple sequence alignments, Hidden Markov Models and molecular modeling. All this makes BACTIBASE a truly unique resource for bacteriocins.

### Future directions

We are currently developing a system for automatic updating of the database. New types of data will be added in the near future. Subsequent development will include integrating a system that automates the prediction of bacteriocin functional amino acids as well as enriching the platform with useful tools for bacteriocin characterization. We also hope to develop new methods/techniques for structural and functional classification of bacteriocins.

## Conclusion

The purpose of the database is to serve the research community by organizing information relevant to all types of bacteriocins from all groups of Bacteria. With the inclusion of most known bacteriocin sequences, BACTIBASE 2 has grown into an integrated knowledge base for bacteriocin investigators. It is our hope that the implementation of 'Molecule Authorities' will transform BACTIBASE into a community-driven database (via notes) and that this trend will continue so that the individual investigators will verify or contribute to verifying every entry. We thank all investigators who have provided or will provide valuable feedback regarding the individual entries in this database. As more information about bacteriocins becomes available, the database will be expanded and improved accordingly. While database updating and developments continue, we welcome your comments, suggestions, or corrections.

## Availability and requirements

BACTIBASE can be accessed freely at http://bactibase.pfba-lab-tun.org.

## Authors' contributions

RH conceived the study, developed the database and web interface and performed the statistical analysis. AZ participated in the design of the study. CLL helped RH annotate sequences and compile the microbiological and physicochemical data. JBH and IF jointly coordinated the project and IF refined the manuscript drafted by RH. All authors read and approved the final manuscript.

## Supplementary Material

Additional file 1 **Table S1**. Distribution, average net charge and amino acid contents of bacteriocins by organism grouping in the BACTIBASE database. Click here for file
