# Supplementary material for: BACTIBASE second release: a database and tool platform for bacteriocin characterization
Source: BMC Microbiol. 2010 Jan 27;10:22. doi: 10.1186/1471-2180-10-22 (PMC2824694; doi:10.1186/1471-2180-10-22)
Supplement: Additional file 1 — Table S1. Distribution, average net charge and amino acid contents of bacteriocins by organism grouping in the BACTIBASE database. [file 1471-2180-10-22-S1.DOC]

## Table 1: Distribution, average net charge and amino acid contents of bacteriocins by organism grouping in the BACTIBASE database

|  | **Count** | **%** | **Charge** | **Basic** | **Acidic** | **Hydrophobic** | **Polar** | **Glycine** | **Cysteine** | **Common** | **Absent** |
| --- | --- | --- | --- | --- | --- | --- | --- | --- | --- | --- | --- |
| **Bacteria** | 177 | 100 | 3.09±4.62 | 8.34±16.78 | 5.25±15.10 | 23.37±40.33 | 24.60±31.85 | 7.66±8.80 | 2.03±1.70 | GA | - |
| **Gram-positive** | 155 | 87.57 | 2.99±2.97 | 5.22±5.49 | 2.22±5.20 | 15.30±14.29 | 18.11±15.14 | 5.99±5.58 | 2.12±1.64 | G | - |
| *Actinoplanes* | 1 | 0.56 | -1 | 0 | 1 | 7 | 11 | 2 | 4 | C | DFHKMNPQRY |
| *Bacillus* | 15 | 8.47 | 0.93±2.55 | 2.20±2.43 | 1.27±0.88 | 10.00±5.79 | 13.80±7.55 | 3.93±5.26 | 3.20±1.93 | G | - |
| *Brevibacterium* | 1 | 0.56 | -13 | 26 | 39 | 101 | 76 | 24 | 0 | AL | C |
| *Brochothrix* | 1 | 0.56 | 2 | 4 | 2 | 21 | 20 | 15 | 1 | G | EMNQRW |
| *Carnobacterium* | 12 | 6.78 | 2.83±1.03 | 4.17±1.19 | 1.33±0.65 | 14.75±6.88 | 19.42±7.12 | 8.58±3.34 | 1.83±1.34 | G | - |
| *Clavibacter* | 1 | 0.56 | 0 | 1 | 1 | 7 | 12 | 2 | 4 | C | DFHKMNPQVY |
| *Clostridium* | 2 | 1.13 | -3.00±1.41 | 5.00±5.66 | 8.00±4.24 | 21.00±5.66 | 10.50±2.12 | 1.50±2.12 | 1.00±1.41 | LE | HW |
| *Enterococcus* | 22 | 12.43 | 4.09±2.88 | 7.73±6.90 | 3.64±4.83 | 20.09±16.90 | 25.18±22.88 | 9.50±8.63 | 2.23±1.54 | G | - |
| *Geobacillus* | 1 | 0.56 | 1 | 4 | 3 | 44 | 23 | 8 | 0 | AI | CHNPY |
| *Kocuria* | 1 | 0.56 | 1 | 2 | 1 | 7 | 12 | 2 | 3 | S | ADKLRWY |
| *Lactobacillus* | 39 | 22.03 | 3.90±2.59 | 5.97±6.93 | 2.08±6.79 | 16.77±15.62 | 17.90±18.13 | 6.64±5.21 | 1.21±1.36 | GA | - |
| *Lactococcus* | 15 | 8.47 | 3.40±1.68 | 5.00±2.17 | 1.60±1.50 | 12.47±7.02 | 15.87±4.53 | 3.80±1.90 | 2.33±2.09 | GKT | - |
| *Leuconostoc* | 5 | 2.82 | 4.60±1.34 | 5.40±0.89 | 0.80±0.45 | 10.60±0.89 | 19.00±5.48 | 7.20±1.79 | 1.60±0.89 | G | - |
| *Listeria* | 1 | 0.56 | 4 | 5 | 1 | 14 | 22 | 7 | 2 | G | EFHLMPR |
| *Paenibacillus* | 1 | 0.56 | 5 | 5 | 0 | 9 | 16 | 1 | 5 | CKT | DEFHMPQRWY |
| *Pediococcus* | 2 | 1.13 | 6.50±0.71 | 7.50±0.71 | 1.00±0.00 | 11.00±1.41 | 21.00±4.24 | 7.50±0.71 | 3.00±1.41 | G | EFL |
| *Propionibacterium* | 3 | 1.69 | 5.00±5.29 | 13.00±14.11 | 8.00±9.64 | 33.33±21.73 | 39.00±34.39 | 8.67±5.51 | 2.00±2.00 | ATG | - |
| *Ruminococcus* | 1 | 0.56 | 1 | 2 | 1 | 7 | 12 | 2 | 3 | CNT | ADPRY |
| *Staphylococcus* | 8 | 4.52 | 5.38±2.56 | 5.88±2.85 | 0.50±0.76 | 10.75±6.25 | 11.88±3.14 | 2.50±1.20 | 2.88±1.25 | K | - |
| *Streptococcus* | 17 | 9.60 | 2.12±1.22 | 3.18±1.24 | 1.06±0.97 | 11.47±8.40 | 14.24±5.04 | 3.88±3.87 | 2.82±1.29 | G | - |
| *Streptomyces* | 5 | 2.82 | 0.40±0.55 | 1.40±0.55 | 1.00±0.00 | 3.60±0.55 | 11.40±1.52 | 2.00±0.00 | 3.00±0.00 | C | EHIM |
| *Weissella* | 1 | 0.56 | 2 | 4 | 2 | 10 | 7 | 2 | 0 | FKV | CHMTW |
| **Gram-negative** | 19 | 10.73 | 6.11±8.67 | 35.72±41.78 | 29.61±36.14 | 91.94±95.97 | 78.67±68.30 | 22.00±16.17 | 1.17±2.09 | A | - |
| *Butyrivibrio* | 2 | 1.13 | 0.00±2.83 | 2.00±1.41 | 2.00±1.41 | 20.50±19.09 | 13.50±2.12 | 4.00±2.83 | 1.50±2.12 | A | R |
| *Escherichia* | 11 | 6.21 | 7.73±9.75 | 43.27±43.90 | 35.55±38.79 | 102.82±89.17 | 87.36±61.54 | 24.09±11.23 | 0.82±1.33 | A | - |
| *Klebsiella* | 1 | 0.56 | -3 | 1 | 4 | 28 | 41 | 19 | 0 | G | CFKR |
| *Myxococcus* | 1 | 0.56 | -1 | 0 | 1 | 10 | 32 | 5 | 8 | CT | EHKMQR |
| *Pseudomonas* | 2 | 1.13 | 12.50±3.54 | 79.50±10.61 | 67.00±7.07 | 236.50±12.02 | 181.50±10.61 | 50.50±3.54 | 0.00±0.00 | A | C |
| *Rhizobium* | 1 | 0.56 | 0 | 1 | 1 | 3 | 5 | 3 | 1 | G | EFHKLMNPTWY |
| *Serratia* | 1 | 0.56 | 2 | 3 | 1 | 3 | 2 | 1 | 0 | HV | ACEFIKMNPQSTW |
| **Archaea** | 3 | 1.69 | -10.00±14.73 | 6.33±10.12 | 16.33±24.83 | 31.33±38.89 | 37.67±44.74 | 8.33±10.12 | 2.67±1.15 | DS | - |
| *Halobacterium* | 2 | 1.13 | -1.50±0.71 | 0.50±0.71 | 2.00±0.00 | 9.00±5.66 | 12.00±7.07 | 2.50±0.71 | 3.00±1.41 | ACGS | EHMRW |
| *Haloferax* | 1 | 0.56 | -27 | 18 | 45 | 76 | 89 | 20 | 2 | D | - |
